# Liver stiffness measurement predicts liver-related events in patients with chronic hepatitis C: A retrospective study

**DOI:** 10.1371/journal.pone.0184404

**Published:** 2017-09-07

**Authors:** Ana Zaida Gomez-Moreno, Daniel Pineda-Tenor, Maria Angeles Jimenez-Sousa, Juan Jose Sánchez-Ruano, Tomas Artaza-Varasa, Jose Saura-Montalban, Pablo Ryan, Salvador Resino

**Affiliations:** 1 Servicio de Digestivo, Hospital Virgen de la Salud, Toledo, Spain; 2 Servicio de Laboratorio Clínico, Hospital Universitario de Fuenlabrada, Madrid, Spain; 3 Unidad de Infección Viral e Inmunidad, Centro Nacional de Microbiología, Instituto de Salud Carlos III, Majadahonda, Spain; 4 Servicio de Laboratorio Clínico, Complejo Hospitalario de Toledo, Toledo, Spain; 5 Servicio de Medicina Interna, Hospital Universitario Infanta Leonor, Madrid, Spain; Yonsei University College of Medicine, REPUBLIC OF KOREA

## Abstract

The management of patients with chronic hepatitis C (CHC) depends on their clinical stage. Thus, noninvasive early recognition of patients with CHC at high risk for developing liver-related events (LREs) is important because it ensures optimal preventative management strategies may be employed that can affect the course of CHC disease. Our aim was to determine whether liver stiffness measurement (LSM) in hepatitis C virus (HCV)-infected patients is associated with a risk of LREs, particularly in cirrhotic patients. We carried out a retrospective study on 343 HCV-infected patients stratified according to cirrhosis (LSM<12.5 kPa vs. LSM≥12.5 kPa), and the cirrhotic patient group (LSM≥12.5 kPa) was divided according to risk of esophageal varices (LSM <25 kPa vs. LSM≥25 kPa). For all patients, each incremental unit in the natural logarithm (Ln) of LSM was associated with 14.76 times higher risk of developing LREs (p<0.001). Patients with cirrhosis (LSM≥12.5 kPa) had a higher risk of LREs than patients without cirrhosis (LSM<12.5 kPa) [adjusted hazard ratio (aHR) = 30.97; p<0.001]. When only cirrhotic patients were analyzed (n = 60), each incremental unit in the Ln of LSM was associated with 10.56 times higher risk of developing LREs (p = 0.010). Patients with LSM≥25 kPa had a greater risk for LRE development compared to those with LSM<25 kPa (aHR = 3.65; p = 0.045). The AUROC for predicting the onset of LREs was 0.876 in all patients and 0.729 in cirrhotic patients. In conclusion, LSM was associated with an increased risk of developing LREs in HCV-infected patients, even within the group of cirrhotic patients.

## Introduction

Nearly 200 million people worldwide are chronically infected with hepatitis C virus (HCV), which leads to the development of chronic liver disease or liver-related death [1,2]. However, the long-term impact of HCV infection is highly variable, ranging from minimal histological changes to extensive fibrosis and cirrhosis [3]. Liver cirrhosis is characterized by a compensated stage (stage 4 fibrosis with or without esophageal varices) followed by a decompensated stage, which can involve complications (variceal bleeding, hepatic encephalopathy, ascites, spontaneous bacterial peritonitis, and/or hepatorenal syndrome), hepatocellular carcinoma, liver transplantation, and liver-related death [4,5]. The management of patients with chronic hepatitis C (CHC) depends on their clinical stage [5], since patients with compensated cirrhosis have a much longer median survival time than those with decompensated cirrhosis [6]. Thus, noninvasive early recognition of patients with CHC at high risk for developing liver-related events (LREs) is important because it ensures optimal preventative management strategies may be employed that can affect the course of CHC disease [7]. Additionally, sustained virological response (SVR) with antiviral therapy may reduce the clinical consequences of CHC, but patients with cirrhosis, despite HCV eradication, remain at risk of disease progression [8].

Decompensated cirrhosis appears as a consequence of portal hypertension, which is defined as a pathologic increase in the pressure gradient between the portal vein and the inferior vena cava measured by hepatic venous pressure gradient (HVPG) [9]. Portal hypertension is a prognostic measure for developing liver decompensation and hepatocellular carcinoma [10,11]. However, measuring the HVPG involves an invasive test that is not widely available. Therefore, noninvasive tests for estimating risk of LREs are needed.

Liver stiffness measurement (LSM) using transient elastography is a noninvasive method based on liver elasticity observations. LSM may accurately predict the presence of advanced fibrosis/cirrhosis and portal hypertension in patients with CHC via the detection of esophageal varices [12–17]. Furthermore, LSM may also predict the future development of LREs in patients with compensated cirrhosis of varying etiologies [18,19]. However, there are few results with a certain degree of discrepancy found in samples from CHC patients, possibly due to the characteristics of the studies carried out. Perez-Latorre et al. found that LSM accurately predicts LREs in HIV-infected patients with CHC [20,21]. Sultanik et al. found that LSM is not a valid surrogate of disease progression of HCV cirrhotic patients in a larger and heterogeneous sample including HIV/HCV-coinfected patients and patients with SVR [22]. Lee et al. found LSM at SVR is useful for predicting LRE development in CHC patients who achieve SVR after treatment with pegylated-interferon-alpha (peg-IFN) plus ribavirin [23]. Fernández-Montero et al. found LSM reliably predicts LREs in HIV/HCV-coinfected patients on antiretroviral therapy [24].

The aim of our study was to determine whether LSM in HCV-infected patients is associated with the risk of developing LREs, particularly in cirrhotic patients.

## Patients and methods

### Patients

We carried out a retrospective study on 343 HCV-infected patients who had liver stiffness assessed by transient elastometry in Hospital Virgen de la Salud (Toledo, Spain) between 2008 and 2014. The study ran from the day of the LSM (baseline) until the last follow-up visit or death, or the initiation of antiviral treatment for HCV. The administrative censoring date was December 31, 2015. This work was conducted in accordance with the Declaration of Helsinki. The Institutional Review Board and the Research Ethic Committee (“Comité de Ética de la Investigación”) of the Instituto de Salud Carlos III approved the study. Each participating patient signed an informed consent form.

The selection criteria were: 1) detectable HCV RNA by polymerase chain reaction (PCR) during the follow-up; 2) availability of a valid baseline LSM; and 3) a follow-up period of at least 12 months after baseline LSM. The exclusion criteria were: 1) co-infection with hepatitis B virus or human immunodeficiency virus; 2) clinical evidence of hepatic decompensation at enrollment or a prior history of hepatic decompensation; and 3) hepatocellular carcinoma at enrollment or a previous history of hepatocellular carcinoma.

### Clinical and laboratory data

Clinical and epidemiological data were obtained from medical records. High alcohol intake was considered to be >20 grams/day in women and ≥60 grams/day in men [25]. Patients received conventional CHC management during follow-up. Thus, patients could have been treated before or after entering the study with HCV therapy according to clinical guidelines [26,27]. The follow-up was truncated, when the HCV therapy was initiated, only whether HCV was cleared, because HCV clearance may lead to regression of liver fibrosis and cirrhosis [28]. When a patient did not achieve SVR, the follow-up was continued.

Analyzed LREs included ascites, hepatic encephalopathy, variceal bleeding, hepatocellular carcinoma, spontaneous bacterial peritonitis and hepatorenal syndrome. Ascites and spontaneous bacterial peritonitis were confirmed or excluded by paracentesis and/or ultrasound. Hepatic encephalopathy and hepatorenal syndrome were established based on clinical findings and laboratory parameters. The source of gastroesophageal variceal bleeding was confirmed by endoscopy. Diagnosis of hepatocellular carcinoma was based on noninvasive imaging tests or histopathology findings [29]. Surveillance or screening for hepatocarcinoma in cirrhotic patients was performed semiannually by abdominal ultrasonography with experienced personnel (hepatologists), as well as the determination of tumor biomarkers (alpha-fetoprotein) [29]. There was no clinical or analytical suspicion of existence of hepatocarcinoma at the time of inclusion in the study.

All the information related to death was reviewed by AZG, who classified death as either: i) CHC-related death, when the series of events that ended in death was caused by a CHC-related complication; or ii) non-CHC-related death.

### HCV assays

HCV infection was documented in all patients by enzyme-linked immunosorbent assay and PCR test. HCV genotype was determined by hybridization of biotin-labeled PCR products to oligonucleotide probes bound to nitrocellulose membrane strips (INNO-LiPA HCV II, Innogenetics, Ghent, Belgium). Plasma HCV RNA viral load was measured by real-time PCR (COBAS AmpliPrep/COBAS TaqMan HCV test), and results were reported in terms of international units per milliliter (IU/mL). The limit of detection was 15 IU/mL.

### Liver stiffness measurement

LSM was assessed by transient elastography (FibroScan^®^, Echosens, Paris, France) using a single machine. Results were expressed in kilopascals (kPa) with a range of 2.5 to 75 kPa [30]. Transient elastography was performed in our unit by a trained hepatologist, and measurements were considered reliable when the interquartile-range-to-median ratio for at least 10 successful measurements was lower than 0.30 [31].

### Outcome and predictor variables

The primary outcome variable was the occurrence of a LRE, defined as the development of a hepatic decompensation (ascites, variceal bleeding, spontaneous bacterial peritonitis, hepatic encephalopathy, and hepatorenal syndrome), hepatocellular carcinoma or liver-related death. For patients who had more than one liver-related event, only the first was included in the analysis of LRE occurrence.

The primary predictor variable was the LSM examined as a continuous variable and a categorical variable. We used established cut-offs of LSM such as <7.1 kPa (F0-F1), 7.1–9.4 kPa (F2), 9.5-12.4 kPa (F3 (bridging fibrosis)), and ≥12.5 kPa (F4 (cirrhosis)) [12]; and ≥25 kPa (predictive of the development of esophageal varices) [32]. In our study, patients were stratified as follows: LSM <12.5 kPa (F0-F3) and LSM ≥12.5 kPa (F4), with the last group divided into LSM <25 kPa and LSM ≥25 kPa.

### Statistical analysis

The statistical analysis was performed with the Statistical Package for the Social Sciences (SPSS) 21.0 (SPSS INC, Chicago, IL, USA). Statistical significance was defined as p<0.05. All p-values were two-tailed.

Values were expressed as absolute number (percentage) and median (25th; 75th percentile). Categorical data and proportions were analyzed using the chi-squared test or Fisher′s exact test. Mann-Whitney U test was used to compare data among independent groups.

For survival analyses, the baseline timepoint was defined as the date of transient elastometry examination. The study ran from the day of the LSM (baseline) until the development of LREs, death, initiation of HCV antiviral therapy, or the censoring date. The Kaplan-Meier analysis was used to estimate the cumulative probability of LREs according to LSM strata, and the log-rank test was used for univariate comparisons among groups. We used the Cox regression analysis to test the association between baseline LSM and LREs. Natural logarithmic (Ln) transformation of LSM was used because of a skewed distribution. Each Cox regression test was adjusted by the most significant covariables for each outcome variable, avoiding overfitting of the regression model. We included the LSM (Enter algorithm (forced entry for the LSM)) and the most relevant characteristics by Forward Stepwise algorithm (at each step, factors are considered for removal or entry: a p-value for entry and exit of 0.15 and 0.20, respectively). The covariables used were age, gender, time since HCV diagnosis, HCV genotype, injection drug use, high alcohol intake, HCV antiviral therapy prior to baseline (patients who failed therapy), and HCV antiviral therapy after baseline (patients who started therapy during follow-up).

We also evaluated the diagnostic performance of LSM for predicting the onset of LREs using the receiver operating characteristic (ROC) curve. We calculated the sensitivity (Se), specificity (Sp), positive predictive value (PPV) and negative predictive value (NPV) for each cut-off point.

## Results

### Baseline characteristics of the study population

Table 1 shows the baseline characteristics of 343 HCV-infected patients, 60 of whom were cirrhotic patients (LSM ≥12.5 kPa). In brief, 57.4% were male, the median age was 49.7 years, 18.7% reported a high intake of alcohol, 14.9% were prior injection drug users, 85.5% were infected by HCV genotype 1. Regarding HCV therapy, 78 (22.7%) patients had failed HCV antiviral therapy (peg-IFN-α/ribavirin) before inclusion in the study, 137 (39.9%) started HCV therapy during follow-up, and of them, 71 (21.1%) patients achieved SVR and their follow-up was truncated. The median LSM baseline was 6.8 kPa and the median time since HCV diagnosis was 9.1 years. When the study population was stratified according to the presence of cirrhosis (LSM >12.5 kPa), cirrhotic patients were older (p = 0.008), had higher time since HCV diagnosis (p = 0.021), and higher percentage of high alcohol intake (p = 0.034) and antiviral therapy during follow-up (p<0.001) than patients without cirrhosis (LSM ≤12.5 kPa).

**Table 1:** Epidemiological and clinical characteristics of HCV-infected patients at baseline.

Baseline characteristics	All patients	F0-F3 (<12.5 kPa)	F4 (≥12.5 kPa)	p-values
**No.**	343	283	60	-
**Male sex**	197 (57.4%)	159 (56.2%)	38 (63.3%)	0.309
**Age (years)**	49.7 (43.5; 57.9)	48.7 (42.7; 57.4)	52.5 (46.7; 63.1)	0.008
**Time of HCV infection (years)**	9.1 (2.5; 14.1)	8.4 (2.4; 13.7)	12.1 (2.9; 16.1)	0.021
**High alcohol intake**	64 (18.7%)	47 (16.6%)	17 (28.3%)	0.034
**Prior injection drug use**	51 (14.9%)	38 (13.4%)	13 (21.7%)	0.103
**HCV genotype (n = 337)**				
** 1**	288 (85.5%)	241 (86.4%)	47 (81.0%)	0.293
** 2**	2 (0.6%)	2 (0.7%)	0 (0.0%)	0.999
** 3**	25 (7.4%)	19 (6.8%)	6 (10.3%)	0.406
** 4**	21 (6.2%)	16 (5.7%)	5 (8.6%)	0.379
** 5**	1 (0.3%)	1 (0.4%)	0 (0.0%)	0.999
**Prior antiviral therapy (peg-IFN-α/RBV) failed**	78 (22.7%)	61 (21.6%)	17 (28.3%)	0.308
**Antiviral therapy during follow-up**	137 (39.9%)	98 (34.6%)	39 (65.0%)	<0.001
**Antiviral therapy with SVR and follow-up truncated**	71 (21.1%)	55 (19.8%)	16 (27.6%)	0.186
**Liver stiffness (kPa)**	6.8 (5.3; 10.2)	6.3 (5.2; 8.1)	20.0 (14.4; 26.9)	<0.001
** F0-F1 (<7.1 kPa)**	187 (54.5%)	187 (66.1%)	-	-
** F2 (7.1–9.4 kPa)**	58 (16.9%)	58 (20.5%)	-	-
** F3 (9.5–12.4 kPa)**	38 (11.1%)	28 (13.4%)	-	-
** F4.1 (12.5–24.9 kPa)**	40 (11.7%)	-	40 (66.7%)	-
** F4.2 (≥25 kPa)**	20 (5.8%)	-	20 (33.3%)	-

Values are expressed as median (p25th; p75th) and absolute count (percentage).

Abbreviations: HCV, hepatitis C virus; kPa, kilopascal.

### Follow-up characteristics of the study population

LREs during follow-up are shown in Table 2. The median follow-up was lower in cirrhotic patients (p = 0.001). Twelve patients experienced one or more LREs; the first LREs were: 8 hepatic decompensations (ascites (n = 6) and variceal bleeding (n = 2)) and 4 hepatocellular carcinomas. Overall, 22 LREs were counted during follow-up: 10 hepatic decompensations [Med = 2.7 years (Min = 0.3; Max = 7.4)], 6 hepatocellular carcinomas [Med = 4.9 years (Min = 2.0; Max = 7.5)], and 6 deaths [Med = 3.4 years (Min = 1.9; Max = 7.6)]. Additionally, throughout the study period, only one patient, who was addicted to drugs, died of septic shock (a cause unrelated to CHC) and this event was not included in the analysis.

**Table 2:** Clinical characteristics of the HCV-infected patients during follow-up.

Follow-up characteristics	All patients	F0-F3 (<12.5 kPa)	F4 (≥12.5 kPa)	p-values
**No.**	343	283	60	-
**Follow-up (years)**	5.1 (2.9; 6.9)	5.5 (3.1; 7.0)	3.7 (2.1; 5.5)	0.001
**Liver-related events (first LRE to appear)**	12 (3.5%)	2 (0.7%)	10 (16.7%)	<0.001
**Hepatic decompensation**	8 (2.3%)	0 (0.0%)	8 (13.3%)	<0.001
** Hepatocellular carcinoma**	4 (1.2%)	2 (0.7%)	2 (3.3%)	0.285
** Death**	0 (0.0%)	0 (0.0%)	0 (0.0%)	-
**Liver-related events (All)**	22 (6.4%)	4 (1.4%)	18 (30.0%)	<0.001
** Hepatic decompensation**	10 (2.9%)	1 (0.4%)	9 (15.0%)	<0.001
** Hepatocellular carcinoma**	6 (1.7%)	2 (0.7%)	4 (6.7%)	0.010
** Death**	6 (1.7%)	1 (0.4%)	5 (8.3%)	0.001

Values are expressed as median (p25th; p75th) and absolute count (percentage).

Abbreviations: HCV, hepatitis C virus; kPa, kilopascal; LRE, liver-related events.

### Liver stiffness measurement and liver-related events

**Fig 1:**
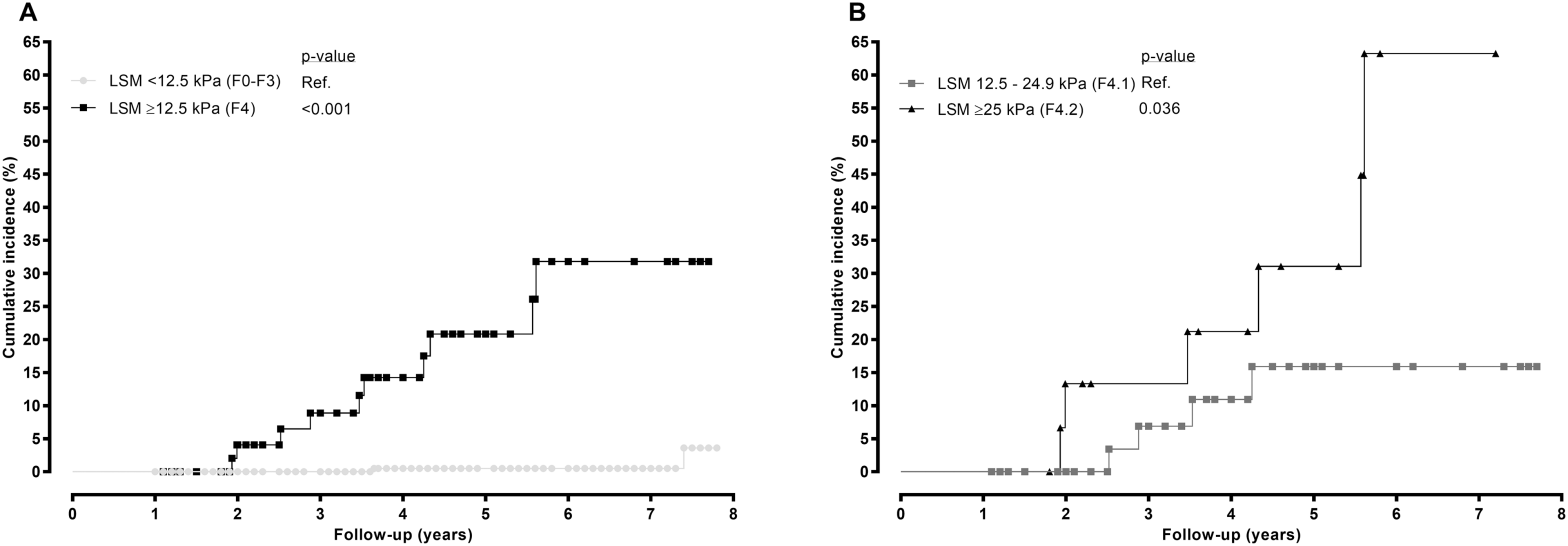
Kaplan-Meier plots showing cumulative incidence of liver-related events according to strata of baseline liver stiffness in HCV-infected patients. P-values were calculated by log-rank test. Abbreviations: kPa, kilopascal; LSM, liver stiffness measurement. (A), All patients stratified by 12.5 kPa. (B), Cirrhotic patients stratified by 25 kPa.

The incidence of LREs during follow-up is shown in Fig 1. Out of 343 patients included in our study, 283 (81.5%) had LSM<12.5 kPa, of which only two developed a LRE (0.6%), whereas 60 (17.5%) patients had LSM≥12.5 kPa and 10 of them had a LRE (16.6%). Kaplan-Meier estimates showed that the probability of LREs was significantly higher in patients with cirrhosis (LSM ≥12.5 kPa) in comparison with patients without cirrhosis (LSM <12.5 kPa) (Fig 1A; p<0.001). Moreover, out of 60 patients with cirrhosis (LSM ≥12.5 kPa), 40 (66.6%) patients had LSM <25 kPa and four of these had a LRE (10%); whereas 20 (33.3%) patients had LSM ≥25 kPa, six of whom had a LRE (30%). The probability of developing LREs was higher in patients with LSM ≥25 kPa than patients with LSM <25 kPa (Fig 1B; p = 0.036).

**Fig 2:**
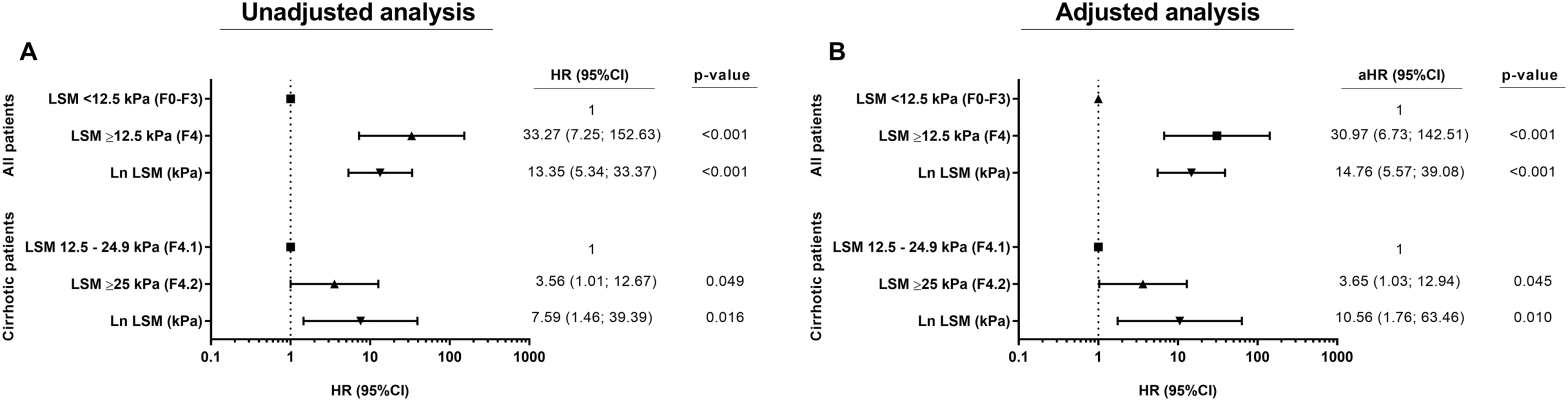
Hazard of liver-related events in HCV-infected patients (Cox proportional hazard regression analysis). Abbreviations: kPa, kilopascal; HR, hazard ratio; aHR, adjusted hazard ratio; 95%CI, 95% of confidence interval; Ln, natural logarithm; LSM, liver stiffness measurement. (A), All patients stratified by 12.5 kPa. (B), Cirrhotic patients stratified by 25 kPa.

Fig 2 shows the risk for the onset of LREs by proportional hazard models, both the unadjusted (A) and the adjusted (B) analysis. For adjusted analysis with all patients (Fig 2B), each incremental unit in the Ln of LSM was associated with 14.74 times higher risk of developing LREs during follow-up [adjusted hazard ratio (aHR) = 14.76; p<0.001]. Furthermore, patients with cirrhosis (LSM ≥12.5 kPa) had a higher risk of developing LREs than patients without cirrhosis (LSM <12.5 kPa) (aHR = 30.97; p<0.001). When only cirrhotic patients were analyzed, each incremental unit in the Ln of LSM was associated with 10.56 times higher risk of developing LREs during follow-up (aHR = 10.56; p = 0.010), and patients with LSM ≥25 kPa had higher risk of LREs than patients with LSM <25 kPa (aHR = 3.65; p = 0.045). The details of the multivariate analysis are shown in the Supporting Information files (S1, S2, S3 and S4 Tables).

### Diagnostic value of liver stiffness measurement for predicting the onset of liver-related events

**Fig 3:**
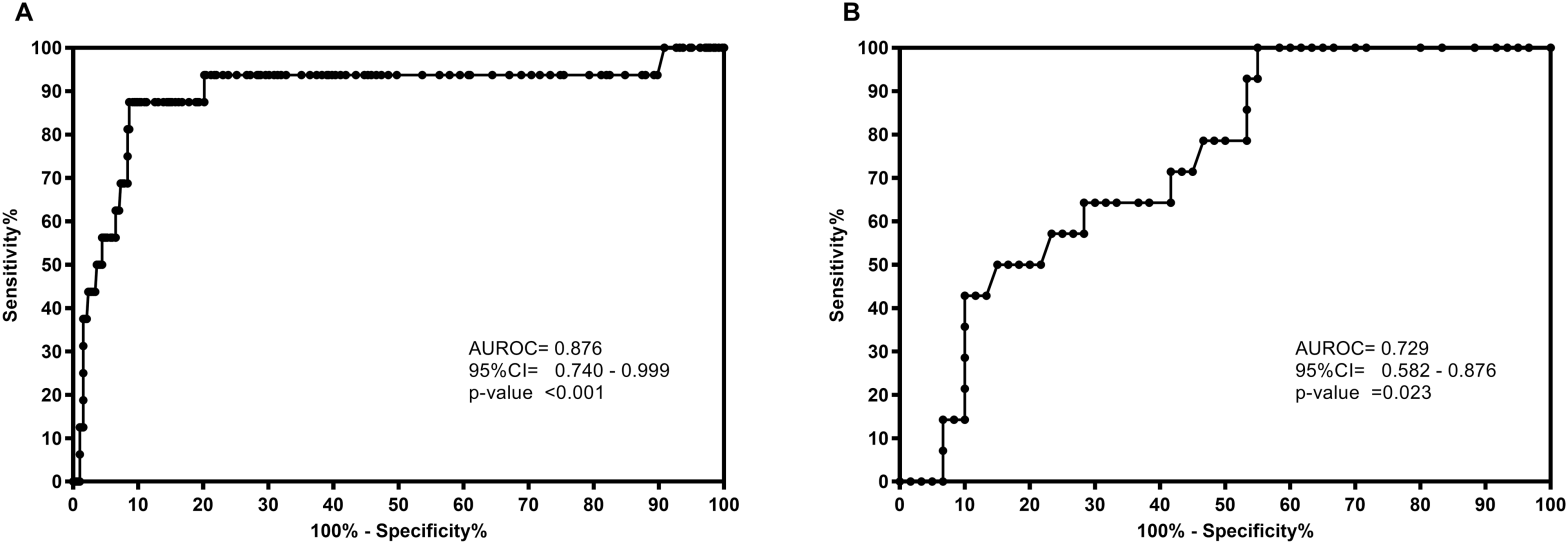
Area under the receiver operating characteristic (AUC-ROCs) curves of liver stiffness for predicting liver-related events in HCV-infected patients. Abbreviations: AUC-ROCs, area under the receiver operating characteristic; 95%CI, 95% of confidence interval. (A), All patients. (B), Cirrhotic patients (LSM≥ 25 kPa).

The area under ROC curve (AUROC) of LSM for predicting the onset of LREs was 0.876 (Fig 3A). Using the cut-off of 12.5 kPa, 10 patients were correctly identified (true positives) as individuals at risk of developing a LRE and only two were misclassified (false negatives) (Table 3A). The NPV were 99.3%, but PPV was under 20%, possibly due to the low prevalence of LREs in our cohort. When the analysis was performed in cirrhotic patients, the AUROC was 0.729 (Fig 3B). Using the cut-off 25 kPa, six patients were correctly identified (true positives) and four were misclassified (false negatives) (Table 3B). The NPV was 90%, but PPV was 30%.

**Table 3:** Diagnostic accuracy of liver stiffness (kPa) measurement for predicting liver-related events in our cohort.

Cut-off	TP	FP	TN	FN	Se	95%CI	Sp	95%CI	PPV	95%CI	NPV	95%CI
**A) All patients**												
** 12.5 kPa**	10	50	281	2	83.3%	55.2% - 95.3%	84.9%	80.6% - 88.4%	16.7%	9.3% - 28.0%	99.3%	97.5% - 99.8%
**B) Cirrhotic patients**												
** 25 kPa**	6	14	36	4	60.0%	31.3% - 83.2%	72.0%	58.3% - 82.5%	30.0%	14.5% - 51.9%	90.0%	76.9% - 96.0%

Abbreviations: kPa, kilopascal; 95%CI, 95% of confidence interval; TP, true positive cases (correct diagnosis); FP, false positive cases (over-diagnosis); TN, true negative cases (correct diagnosis); FN, false negative cases (missed cases); Se, sensitivity; Sp, specificity; PPV, positive predictive value; NPV, negative predictive value.

## Discussion

Staging of liver fibrosis is an essential element of the care of patients with CHC, as it provides useful prognostic data and facilitates treatment decisions [4,5]. Given the importance of assessing the risk of LREs in the management of CHC patients, we evaluated whether LSM may predict the occurrence of LREs in CHC patients, finding that patients with higher LSM had a higher risk of developing LREs. Moreover, this association was maintained in cirrhotic patients (LSM ≥12.5 kPa), where patients with LSM ≥25 kPa had a higher risk of developing LREs than patients with LSM <25 kPa. These findings may be clinically relevant, particularly since the use of transient elastography in the clinical management of this population is increasing. Moreover, since there have been recently reported that the incidence of hepatocellular carcinoma has increased even after treatment of chronic hepatitis C [33–36], the results of this study also highlight the importance of monitoring the liver stiffness in CHC patients in the future.

The association found between higher LSM and higher risk of developing LREs is consistent with other reports [19,21,37]. In our study, we observed that a high LSM, independently of other epidemiological and clinical factors related to liver disease severity, was able to predict future risk of LREs (liver decompensation or hepatocellular carcinoma) in a direct relationship manner. Each incremental Ln unit of LSM was associated with 14.76 times higher risk of LREs in all patients and 10.56 times higher in cirrhotic patients. Furthermore, patients with liver cirrhosis (≥12.5 kPa) had a higher risk of developing LREs; and within that group, patients with decompensated cirrhosis (LSM ≥25 kPa) had an increased risk of LREs compared to patients with compensated cirrhosis (LSM 12.5–24.9 kPa). Thus, patients with LSM ≥12.5 kPa, and particularly those with LSM >25 kPa, would benefit from a greater degree of monitoring for hepatic complications. Conversely, patients with LSM <12.5 kPa might be safely reassured of a low risk of hepatic complications during the following several years. The cut-off 12.5 kPa could be used to defer close surveillance in patients with low liver stiffness values, providing reassurance and minimizing healthcare expenditure.

The increased risk of LREs according to liver stiffness categories suggests that LSM offers prognostic information beyond that provided by liver biopsy. Whereas the cirrhosis stage is defined by histological evidence with one or two qualitative categories (e.g. METAVIR stage 4 or Ishak stages 5 and 6), the dynamic range of LSM is much greater (from 12.5 to 75 kPa in cirrhosis) and it has shown that LSM has greater diagnostic performance than liver biopsy for predicting hepatic complications [38,39]. In our study, LSM values were accurate for the prediction of the development of a first LRE, since the AUROC value was above 0.85 for all patients and somewhat lower for cirrhotic patients (AUROC = 0.729), which supports its excellent discriminatory ability. Our AUROC data were similar or better than those previously published [21,38,39]. Furthermore, our Se, Sp, PPV and NPV values were also similar to those previously reported [21,37–39]. The cut-offs evaluated in our study showed NPV values higher than 90%, which could be acceptable for excluding the onset of LREs; whereas the PPV values were around 30%, which could be unacceptable for predicting the first LREs. Therefore, the practical value of these cut-offs for assessing the onset of LREs might be important, as they will give very few false negatives, whereas these cut-offs could also result in a lot of false positives, which would undergo a more thorough follow-up due to a high LSM.

This study has other limitations that must be taken into account to ensure correct interpretation of the data. Firstly, this is a retrospective study and, therefore, patients are selected beforehand from among subjects surviving long enough to yield sufficient follow-up. However, the assessment of the longitudinal association between baseline LSM and LREs is the strength of this analysis. Secondly, our study is limited by its sample size and the small number of events recorded, which may have impaired the ability to detect robust associations and the accuracy of the risk estimates. Thirdly, about 23% of patients were previously treated against HCV infection, before baseline, but they did not remove HCV infection. The first studies already showed beneficial effects in virological nonresponders shortly after cessation of treatment, but not during long-term follow-up [40–42]. Later, other studies been reported that antiviral treatment failure does not appear to affect the natural course of CHC during long-term follow-up [43,44].

## Conclusions

In conclusion, our findings indicate that LSM was associated with an increased hazard of developing the first episode of LREs in HCV-infected patients and, then, LSM may be a valid noninvasive test for stratifying the risk for developing LREs in patients with CHC.

## Supporting information

S1 Table Supporting information. Hazard of liver-related events in HCV-infected patients according to the liver stiffness measurement (LSM). Data were analyzed by multivariate Cox regression analysis using Forward Stepwise algorithm.
(XLSX)

S2 Table Supporting information. Hazard of liver-related events in HCV-infected patients according to the cut-off of liver stiffness measurement (LSM) 12.5 kPa. Data were analyzed by multivariate Cox regression analysis using Forward Stepwise algorithm.
(XLSX)

S3 Table Supporting information. Hazard of liver-related events in cirrhotic HCV-infected patients according to the liver stiffness measurement (LSM). Data were analyzed by multivariate Cox regression analysis using Forward Stepwise algorithm.
(XLSX)

S4 Table Supporting information. Hazard of liver-related events in cirrhotic HCV-infected patients according to the cut-off of liver stiffness measurement (LSM) 12.5 kPa. Data were analyzed by multivariate Cox regression analysis using Forward Stepwise algorithm.
(XLSX)
